# Silencing of Phytopathogen Communication by the Halotolerant PGPR *Staphylococcus Equorum* Strain EN21

**DOI:** 10.3390/microorganisms8010042

**Published:** 2019-12-24

**Authors:** Clara Vega, Miguel Rodríguez, Inmaculada Llamas, Victoria Béjar, Inmaculada Sampedro

**Affiliations:** 1 Department of Microbiology, Faculty of Pharmacy, University of Granada, 18071 Granada, Spain; cvegazayas@gmail.com (C.V.); miguelrg@correo.ugr.es (M.R.); vbejar@ugr.es (V.B.); 2 Institute of Biotechnology, Biomedical Research Center (CIBM), University of Granada, 18100 Granada, Spain

**Keywords:** *Staphylococcus equorum* strain EN21, halotolerant bacterium, bacterial phytopathogen, PGPR, quorum sensing, quorum quenching, communication silencing

## Abstract

Increasing world food demand together with soil erosion and indiscriminate use of chemical fertilization highlight the need to adopt sustainable crop production strategies. In this context, a combination of plant growth-promoting rhizobacteria (PGPR) and pathogen management represents a sustainable and efficient alternative. Though little studied, halophilic and halotolerant PGPR could be a beneficial plant growth promotion strategy for saline and non-saline soils. The virulence of many bacterial phytopathogens is regulated by quorum sensing (QS) systems. Quorum quenching (QQ) involves the enzymatic degradation of phytopathogen-generated signal molecules, mainly N-acyl homoserine lactones (AHLs). In this study, we investigate plant growth-promoting (PGP) activity and the capacity of the halotolerant bacterium *Staphylococcus equorum* strain EN21 to attenuate phytopathogens virulence through QQ. We used biopriming and in vivo tomato plant experiments to analyse the PGP activity of strain EN21. AHL inactivation was observed to reduce *Pseudomonas syringae* pv. tomato infections in tomato and *Arabidopsis* plants. Our study of *Dickeya solani*, *Pectobacterium carotovorum* subsp. *carotovorum* and *Erwinia amylovora* bacteria in potato tubers, carrots and pears, respectively, also demonstrated the effectiveness of QS interruption by EN21. Overall, this study highlights the potential of strain *S. equorum* EN21 in plant growth promotion and QQ-driven bacterial phytopathogen biocontrol.

## 1. Introduction

In recent years, the growing food demand worldwide has highlighted the need for sustainable crop production. The principal causes of food scarcity include biotic and abiotic stress conditions such as salinity, drought and plant pathogens [1]. Crop quality and yields are also affected by global climate change, while the shortfall in global agricultural output with regard to the demand for food is alarming (www.fao.org/economic/esa). 

Indiscriminate use of chemical fertilizers and antibiotics in traditional agriculture has become a significant cause of soil and water pollution and other environmental problems [2]. In this context, new strategies, including plant growth promotion and pathogen management techniques to optimise productivity and crop quality, are crucial. Plant growth-promoting rhizobacteria (PGPR) have also been widely recognised as important tools for maintaining root health and nutrient uptake [3]. 

Though poorly studied, halophilic and halotolerant PGPR could be effectively used as part of a plant growth promotion strategy for saline and non-saline soils. These bacteria, whose multiple mechanisms are involved in producing enzymes such as 1-aminocyclopropane-1-carboxylic acid (ACC) deaminase, auxins, exopolysaccharides and siderophores, play an important role in boosting plant growth under stress conditions including salinity [4].

The virulence and associated functions of many bacterial phytopathogens, which cause major economic losses, are regulated by quorum sensing (QS) systems. One promising strategy, involving quorum quenching (QQ), to combat infections is based on the enzymatic degradation of signal molecules [5]. QS is used by a wide variety of bacteria to regulate gene expression in a cell density-dependent manner [6,7,8]. This mechanism involves the production, release and recognition of the accumulation of signalling molecules known as autoinducers [9]. The best-studied autoinducers are *N*-acyl homoserine lactones (AHLs) which are produced by many *Proteobacteria*. Diffusible signal factors (DSFs) and quinolones, which have been identified in *Pseudomonas aeruginosa* [10] and *Xanthomonas* spp. [11], are also autoinducers. QS plays an important role in the regulation of bacterial functions including virulence gene expression, biofilm formation and antibiotic resistance [6,12,13,14,15]. In plant pathogenic bacteria such as *Pseudomonas syringae* pv. *syringae* [12], *Pectobacterium carotovorum* [16] and *Agrobacterium tumefaciens* [17], QS has been reported to regulate cell wall-degrading enzyme production, cellular motility and biofilm formation. Given the contribution of these QS-coordinated virulence factors to the pathogenesis of phytopathogens [14], quorum sensing signal degradation could be an effective phytopathogen biocontrol strategy.

QQ involves AHL-degrading enzymes such as lactonase, acylase and oxidorreductase [18], which have been identified in bacterial species including *Bacillus* spp. [19], *Pseudomonas* spp. [20] and *Rhodococcus* spp. [21]. However, little is known about the role played by bacterial signalling communication in pathogen management. 

Results regarding the reduction of virulence in bacterial plant pathogens, especially with respect to *Pectobacterium carotovorum*-potato soft rot, have been promising [22,23,24,25]. We selected halotolerant strain *Staphylococcus* sp. EN21, previously isolated from the halophyte *Salicornia hispanica,* for its *in vitro* plant growth-promoting properties and its efficient degradation of C10-HSL. We aimed to analyse PGP activity and the ability to attenuate the virulence of phytopathogenic bacteria by QQ of EN21. The study provides information on the PGP activity of this strain which was evaluated with the aid of biopriming assays and *in vivo* experiments with tomato plants. QQ activity was tested in synthetic AHLs and crude AHL extracts from bacterial phytophatogens. Overall, we describe a beneficial strategy to regulate plant bacterial infections in different hosts involving a combination of plant growth promotion and pathogen communication silencing.

## 2. Experimental Procedures

### 2.1. Bacterial Strains, Media, Compounds and Culture Conditions

The strain *Staphylococcus* sp. EN21 selected for this study was previously isolated from the rhizosphere of the *Salicornia hispanica* plant taken from El Saladar del Margen (Cúllar, Granada; 37° 38′43.6″ N, 2°35′59.0″ W). The following plant bacterial pathogens were used—*Agrobacterium fabrum* C58 ATCC 33970^T^, *Dickeya dianthicola* DSM 18054^T^, *D. solani* IPO 2222^T^, *Erwinia amylovora* CECT 222^T^, *Pectobacterium atrosepticum* CECT 314^T^, *P. carotovorum* subsp. *carotovorum* CECT 225^T^, *Pseudomonas syringae* pv. *syringae* LMG 1247^T^ and *P. syringae* pv. tomato DC3000.

Strain EN21 and the phytophatogens were grown in trypticase soy broth (TSB) medium. The biosensor strains *Chromobacterium violaceum* CV026 [26] and *C. violaceum* VIR07 [27] were cultured in Luria-Bertani (LB) medium, while *Agrobacterium tumefaciens* NTL4 (pZLR4) was grown in *Agrobacterium* broth (AB) medium [28]. Malt Extract Yeast Extract (MY) medium [29], supplemented with a sea-salt solution [30] containing 5% (*w*/*v*) NaCl, was used for selective plate counts. All strains were grown during 16 h at 28 °C and 100 rpm on a rotary shaker unless otherwise stated. When needed, antibiotics were used in final concentrations of 50 μg mL^−1^ kanamycin (Km) and 50 μg mL^−1^ gentamicin (Gm). 

The following synthetic AHLs (Sigma^®^) were used—C4-HSL (*N*-butyryl-dl-homoserine lactone), C6-HSL (*N*-hexanoyl-dl-homoserine lactone), 3-O-C6-HSL (*N*-3-oxo-hexanoyl-dl-homoserine lactone), C8-HSL (*N*-octanoyl-dl-homoserine lactone), 3-O-C8-HSL (*N*-3-oxo-octanoyl-dl-homoserine lactone), C10-HSL (*N*-decanoyl-dl-homoserine lactone), 3-OH-C10-HSL (*N*-3-hydroxydecanoyl-dl-homoserine lactone), C_12_-HSL (*N*-dodecanoyl-dl-homoserine lactone) and 3-O-C12-HSL (*N*-3-oxo-dodecanoyl-dl-homoserine lactone).

### 2.2. Characterization of Strain EN21

Optimal growth and salt stress tolerance of EN21 were tested in Tryptic Soy Broth (TSB) medium supplemented with 0–25% (*w*/*v*) NaCl. Other phenotypic characteristic were evaluated—acid and alkaline phosphatase [31,32]; hydrolysis of starch and casein [33], cellulose [34], chitin [35], DNA [36], Tween^®^ 20, Tween^®^ 80 and gelatin [33] and siderophore production [37]. EN21 toxicity was assessed using the Microtox^®^ rapid test system (Environmental Bio-Detection Products Inc., Mississauga, ON, Canada) [38].

Genomic DNA was isolated from strain EN21 using the X-DNA purification kit (Xtrem Biotech S.L., Granada, Spain). The 16S rRNA gene was amplified with the aid of universal bacterial primers 16F27 and 16R1488 and the PCR product was then purified and cloned into the pGEM^®^-T vector (Promega, Fitchburg, WI, USA). Using BLASTN software [39], the DNA sequence obtained was compared to reference 16S rRNA gene sequences in the GenBank and EMBL databases and pairwise 16S rRNA gene sequence similarity was calculated using the EzBioCloud server [40].

### 2.3. Plant Growth-Promoting Assays

The PGP traits of strain EN21 were tested in tomato (*Solanum lycopersicum* L.) seeds and seedlings for biopriming and growth-promoting assays, respectively. In both cases, tomato seeds were surface-sterilised with 2.5% (*w*/*v*) sodium hypochlorite for 15 min, followed by 7% (*v*/*v*) hydrogen peroxide for 15 min. Seeds were washed four times in sterile distilled water and left to imbibe for 90 min in sterile distilled water. For biopriming, sterilised seeds were incubated with a 10^9^ CFU mL^−1^ suspension of strain EN21 cells in water for 1 hour at 28 °C and 120 rpm, with sterile distilled water used as control. Thirty bacterised seeds were placed on each petri dish containing 5 mL sterile distilled water-soaked paper and then incubated for 4 days at 28 °C in darkness. Three replicates were performed per treatment. The effects of biopriming were evaluated using the vigour index (VI) [41]. For the plant growth promotion assay, 10 sterilised seeds were sown in individual pots containing sterilised vermiculite (3 cycles at 121 °C and at 1 atm for 20 min). Each pot was irrigated with 250 µL of 10^9^ CFU mL^−1^ suspension and 250 µL of sterile distilled water (control) weekly for 3 weeks. Pots were kept in an indoor greenhouse during a long-day photoperiod (16:8 h light:dark) at 25 °C for 4 weeks. The plants were then harvested and the aerial and radicular parts were measured and oven-dried at 40 °C for 48 h for subsequent dry weight determination.

### 2.4. Quorum Quenching Activity Against Synthetic AHLs and Crude AHL Extracts From Plant Bacterial Pathogens 

QQ activity of *Staphylococcus* sp. strain EN21 for AHL degradation was analysed using a well diffusion agar-plate assay [42,43]. Briefly, each synthetic AHL in a final concentration of 25 µM was added to overnight cultures of strain EN21 and then incubated at 28 °C for 24 h. Cell-free TSB medium supplemented with the same concentration of AHLs was incubated as negative control. The remaining AHLs were detected on LB agar plates overlaid with *C. violaceum* CV026 to detect short- and medium-chain AHLs, with *C. violaceum* VIR07 to detect C10-HSL and 3-OH-C10-HSL and on AB agar plates supplemented with 80 µg mL^−1^ of 5-bromo-4-chloro-3-indolyl-ß-d-galactopyranoside (X-gal) overlaid with *A. tumefaciens* NTL4 (pZRL 4) to detect medium- and long-chain AHLs. After incubation, AHL production was checked for the appearance of a purple or blue colour around each well.

To determine QQ activity against AHLs produced by phytopathogens, crude AHL extracts from *Agrobacterium fabrum* C58 ATCC 33970^T^, *Dickeya dianthicola* DSM 18054^T^, *D. solani* IPO 2222^T^, *Erwinia amylovora* CECT 222^T^, *Pectobacterium atrosepticum* CECT 314^T^, *P. carotovorum* subsp. *carotovorum* CECT 225^T^, *Pseudomonas syringae* pv. *syringae* LMG 1247^T^ and *P. syringae* pv. tomato DC3000 were collected as described elsewhere [25,44,45]. Briefly, AHLs from an overnight culture of each bacterium were extracted twice with dichloromethane (1:1), then dried and re-suspended in 15 µL of 70% (*v*/*v*) methanol. A crude AHL extract from each pathogen (5 µL) was added to 5 mL of an overnight EN21 culture in LB medium. A similar quantity of each crude extract was added to 5 mL cell-free TSB medium for use as control. After incubation at 28 °C for 24 h at 150 rpm, the remaining AHLs were extracted as described above and 20 µL of each sample was spotted on sterile paper disks (5 mm in diameter), placed on AB agar plates and detected using biosensor *A. tumefaciens* NTL4 (pZLR4). 

### 2.5. Competitive Assay 

The antagonistic activity of strain EN21 from the plant bacterial pathogens used in this study was evaluated according to the well diffusion method [46]. An overlay of each pathogen was prepared on LB agar plates and 100-µL aliquots of the supernatant from a 5-day culture of strain EN21 was placed in the wells previously done. After 48 h of incubation at 28 °C, plates were examined for growth inhibition halos surrounding the wells.

### 2.6. Phytopathogens QS System Interference by Co-Culture Assays 

Co-culture assays of plant bacterial pathogens and strain EN21 were carried out according to the methodology described by Torres et al. [43]. Briefly, 10^9^ CFU mL^−1^ of each pathogenic strain was co-cultured with 10^9^ CFU mL^−1^ of strain EN21 (1:100) in TSB medium at 28 °C and 150 rpm for 24 h. A similar concentration of each pathogen was added to cell-free TSB medium as negative control. The remaining AHLs from each co-culture were extracted as described above and, using the well diffusion agar method, were detected on AB agar plates supplemented with X-gal and overlaid with biosensor *A. tumefaciens* NTL4 (pZRL4). The abundance of each bacterium was determined in mono- and co-cultures by serial dilution and plate counts using Tryptic Soy Agar (TSA) plates in which colony morphology was used to determine inter-species differences. In the case of similar colony patterns, double plate counts were performed using TSA plates and MY agar plates with a 5% (*w*/*v*) NaCl solution as selective medium for strain EN21. To analyse the impact of AHL degradation in the QS system of the phytopathogens, virulence and phenotypic traits were evaluate as described above.

### 2.7. Tuber and Fruit Virulence Assays

The ability of strain EN21 to interfere with soft rot caused by *D. solani* IPO 2222^T^, *P. carotovorum* subsp. *carotovorum* CECT 225^T^ and *Erwinia amylovora* CECT 222^T^ was assessed in potato and carrot slices and pear halves, respectively [24]. Briefly, potatoes (*Solanum tuberosum*), carrots (*Daucus carota*) and pears (*Pyrus communis*) were tap-washed and surface-sterilised by spraying with 1% (*w*/*v*) sodium hypochlorite solution, followed by 70% (*v*/*v*) ethanol and sterile distilled water. The potatoes and carrots were cut into slices (0.5 cm in width), while the pears were cut down the middle under sterile conditions and arranged in petri dishes. The EN21-pathogen co-culture was prepared as described above—5 µL of the mixture were inoculated in the potato slices and pear halves with three equidistant incisions, while the carrot slices were inoculated with a single incision. Mono-cultures of each bacterium, as well as sterile distilled water, were similarly inoculated in potatoes, carrots and pears as controls. Nine replicates of each treatment were performed. After 24–48 h of incubation at 28 °C, the maceration zones were visually detected and the extent of damage was calculated by image analysis using ImageJ software [47]. Co-culture plate counts were performed to verify the concentrations of the pathogen and strain EN21.

### 2.8. In Vivo Arabidopsis Plant Virulence Test

The effect of AHL degradation by strain EN21 on *P. syringae* pv. tomato DC3000 virulence was tested in *Arabidopsis* plants using a technique described by Timmermann et al. [48] with slight modifications. Thus, *Arabidopsis* seeds were surface-sterilised and sown in 96-well plates with Murashige and Skoog medium [49] supplemented with 0.22 µm filter sterilised sucrose to a final concentration of 7.3 mM. Plants were treated with sterile distilled water, the pathogen, strain EN21 and EN21-pathogen co-culture. Chlorophyll content was determined in fresh leaves by acetone extraction and absorbance reading [50,51,52]. Chlorophyll fluorescence images of *Arabidopsis* leaves were recorded under UV excitation (365 nm wavelenght) using a Leica DM5500 B fluorescence microscopy with LED illuminator system.

### 2.9. In Vivo Tomato Plant Virulence Test

The effect of AHL degradation by strain EN21 on *P. syringae* pv. tomato DC3000 virulence was tested in tomato plants using a technique described elsewhere [53]. Thus, tomato seeds were surface-sterilized and sown in pots as previously described in the plant growth promotion assay section. Plants were treated with sterile distilled water, the pathogen, strain EN21 and EN21-pathogen co-culture. Three pots containing 50 tomato seeds per pot were used for each treatment. For plants treated with strain EN21 and the co-culture, a 10 mL suspension of strain EN21 (10^9^ CFU mL^−1^) was inoculated weekly for 3 weeks to enable EN21 cells to interact and to establish plantlets, while the other two treatmentws were inoculated with 10 mL sterile distilled water. The pots were kept in an indoor greenhouse during a long-day photoperiod (16:8 h light:dark) at 25 °C for four weeks and watered with 50 mL sterile distilled water twice a week. After three weeks, the pots were exposed to 100% humidity for 16 h to induce stomatal opening and were then sprayed with a 5 mL *P. syringae* pv. tomato DC3000 suspension (10^9^ CFU mL^−1^). Relative humidity was maintained at 100% for a further 24 h to facilitate pathogen infection. On day seven post-inoculation, affected and unaffected shoots were counted followed by plant harvesting. Shoots and roots were oven-dried to estimate dry weight. One hundred milligrams of fresh shoots were used to determine chlorophyll content as described above. 

### 2.10. Statistical Analysis

Data normality was verified by the Shapiro-Wilk test and the data were statistically analysed with the aid of the ANOVA (*p* ≤ 0.05) and Tukey tests using SPSS 24.0 software. 

## 3. Results

### 3.1. Characterization of Strain EN21

Strain EN21 was isolated from the rhizosphere of *Salicornia hispanica*, which was taken from El Saladar del Margen (Cúllar, Granada, Spain) and was selected from a previous screening of 49 bacteria for their plant growth promotion properties. EN21 produced the enzymes ACC-deaminase, phosphatase and nitrogenase, hydrolysed cellulose and Tween 20, as well as siderophores and surfactants. 

Complete 16S rRNA gene sequencing (1434 bp) confirmed that strain EN21 is a member of the bacterial genus *Staphylococcus equorum,* showing 99.86% identity with strain *S. equorum* PA 231^T^ [54]. Its haloterant properties were attested by its growth in a wide range of salt concentrations (0–25% (*w*/*v*) NaCl) and, according to the Microtox test, does not show toxicity, with an EC_50_ of 125.7%. 

### 3.2. Plant Growth Promotion Capacity of Strain EN21 

To evaluate its PGP activity, we performed a preliminary tomato seed bacterization experiment with *S. equorum* strain EN21. An increase in the shoot and root length of tomato seeds was observed in the inoculation with EN21 as compared to the non-inoculated control (Table 1 and Figure S1). Seed bacterization significantly increased both the germination rate and the vigour index by 15.7% and 24.4%, respectively.

In vivo experiments were carried out to evaluate the PGP activity of strain EN21 in tomato plants. Plant inoculation with the strain tested in this study did not significantly affect growth promotion. However, combining seed bacterization with bacterial inoculation led to a significant improvement in plant growth (Table 2 and Figure S2). This improvement corresponded to longer plant shoots and higher levels of shoot and root dry weight as compared to the non-inoculated and non-bacterised controls (Table 2). We observed an increase of 110.5% in total plant dry weight, thus confirming that strain EN21 boosts plant growth. No statistical differences in root or total plant length in relation to the non-treated controls were observed. 

### 3.3. QQ Activity of EN21 against Synthetic AHLs and Crude AHL Extracts From Pathogenic Bacteria

We studied the capacity of EN21 to degrade a range of synthetic AHLs—C_4_-HSL, C_6_-HSL, 3-oxo-C_6_-HSL, C_8_-HSL, 3-oxo-C_8_-HSL, C_10_-HSL, 3-OH-C_10_-HSL, C_12_-HSL, 3-oxo-C_12_-HSL and C_14_-HSL. A well diffusion agar-plate assay with *Agrobacterium tumefaciens* NTL4 (pZLR4) biosensor strains was used to detect a wide range of AHLs, including *Chromobacterium violaceum* CV026 to detect C_4_-HSL and *C. violaceum* VIR07 to detect C_10_-HSL and 3-OH-C_10_-HSL. *S. equorum* strain EN21 showed an intense QQ activity against the complete range of AHLs tested with no remaining AHLs detection except for C_12_-HSL in which degradation was not observed. 

**Figure 1:**
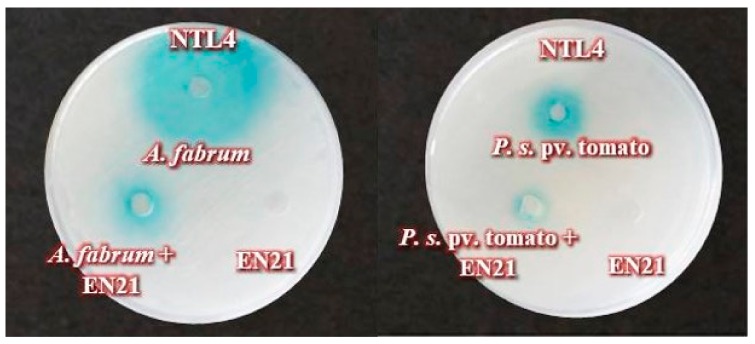
Quorum quenching (QQ) activity of strain EN21 against crude N-acyl homoserine lactone (AHL) extracts from *A. fabrum* and *P. syringae.* pv. tomato.

In addition, we evaluated the QQ activity of EN21 against crude AHL extracts from the following eight pathogenic strains—*Agrobacterium fabrum* C58 ATCC 33970^T^, *Dickeya dianthicola* DSM 18054^T^, *D. solani* IPO 2222^T^, *Erwinia amylovora* CECT 222^T^, *Pectobacterium atrosepticum* CECT 314^T^, *P. carotovorum* subsp. *carotovorum* CECT 225^T^, *Pseudomonas syringae* pv. *syringae* LMG 12472^T^ and *P. syringae* pv. tomato DC3000. Our results indicate that EN21 is, to a greater or lesser extent, capable of degrading AHLs produced by all the pathogens tested except for *D. dianthicola* which AHLs were not affected by EN21 strain. A strong AHLs degradation was achieved for *A. fabrum, D. solani*, *P. s.* pv. *syringe* and *P. s.* pv. tomato, while partial degradation was reached for *E. amylovora, P. atrosepticum* and *P. carotovorum* subsp. *carotovorum*. Figure 1 shows the significant degradation of certain AHLs caused by EN21. 

### 3.4. Attenuation of Phytopathogenic Virulence Factors in Co-Cultures with EN21

EN21-phytopathogen co-cultures were used to analyse the repercussion of AHLs degradation on phytopathogens virulence attenuation. Using antagonist experiments, we first confirmed that EN21 did not interfere with growth in any of the eight plant pathogens tested. Each pathogen was grown in a co-culture with EN21 (ratio 1:100). After 24 h of incubation, the remaining AHLs were detected using the biosensor *A. tumefaciens* NTL4 (pLZR4). All AHLs produced by each pathogen, except for *D. dianthicola,* were partially degraded, which is in line with our previous findings. The same co-cultures were used to analyse the effect of AHL degradation on QS-regulated cellular functions. In the co-cultures with EN21, we observed a reduction in enzymatic activities such as caseinase, DNase and lecithinase in *D. solani* IPO 2222^T^, as well as amylase in *P. syringae* pv. *syringae* LMG 1247^T^ and *P. syringae* pv. tomato DC3000 (Table S1). 

### 3.5. EN21 Enhances Disease Resistance to Phytopathogens in Tubers and Fruits

**Figure 2:**
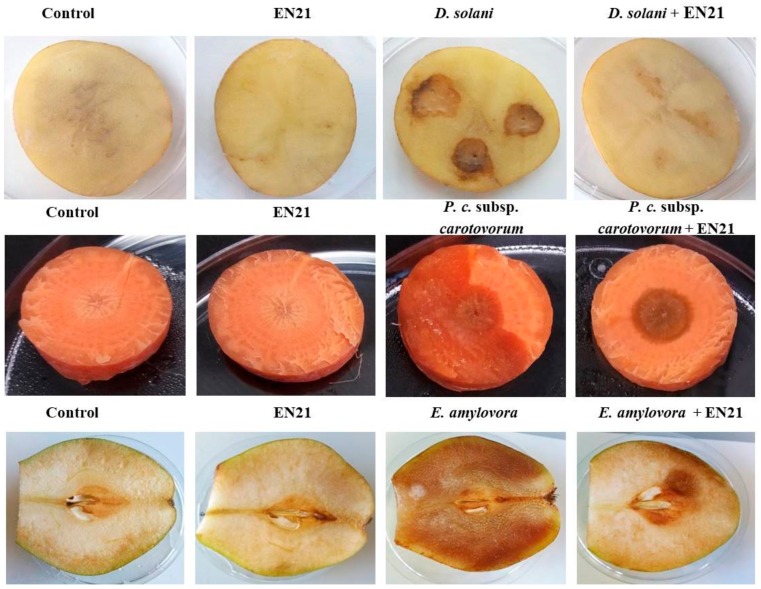
Effect of EN21 AHL-degrading activity against bacterial plant pathogens in potato tuber, carrot and pear assays. Tissues were inoculated with sterile water (control), EN21 and phytopathogen mono-cultures and phytopathogen-EN21 co-cultures (from left to right in panel).

We carried out experiments on potatoes, carrots and pears which demonstrated the effectiveness of QS interruption by EN21 against *D. solani* IPO 2222^T^, *P. carotovorum* subsp. *carotovorum* CECT 225^T^ and *Erwinia amylovora* CECT 222^T^, respectively. Bacterial mono-cultures and EN21-pathogen co-cultures were prepared under the conditions described above. Potato tubers infected with the *D. solani* IPO 2222^T^ mono-culture produced large maceration zones (25%), while a reduction in soft rot symptoms (5% maceration) was observed in the pathogen-EN21 co-culture (Figure S3A and Figure 2). When inoculated with the EN21-*P. carotovorum* subsp. *carotovorum* CECT 225^T^ co-culture, the carrot slices showed less maceration (Figure S3B and Figure 2). QS interruption by EN21 was highly effective in pears, with almost a 90% reduction in maceration caused by *E. amylovora* CECT 222^T^-induced fire blight in the EN21-pathogen co-culture (Figure S3C and Figure 2). The concentrations of strain EN21 and each pathogen, measured by plate counting, were found to be similar throughout the experiment. Thus, strain EN21 proved to be capable of biologically controlling different host and phytopathogen types.

### 3.6. EN21 Reduces P. syringae pv. Tomato DC3000 Virulence in Model Plant Arabidopsis 

**Figure 3:**
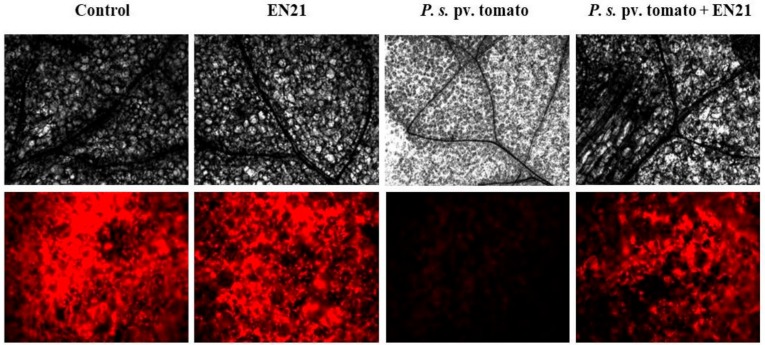
Visualization of tissue structure and chlorophyll content in *Arabidopsis* leaves by differential interference contrast (DIC) and epifluorescence microscopy 100× (first and second row, respectively). Leaves were inoculated with sterile water (control), EN21 and phytopathogen mono-cultures and phytopathogen-EN21 co-culture (from left to right in panel). Micrographs from each row were taken under the same exposure time, gamma and gain values for comparative analysis. Epifluorescence images were digitally coloured.

Disease spread caused by *P. syringae* pv. tomato DC3000 in *Arabidopsis* plants was monitored by visually inspecting leaf tissue chlorosis at 3 dpi. We used microscopic monitoring to assess *Arabidopsis* plant responses to this phytopathogen in the presence and absence of strain EN21. The pathogen was inoculated and disease symptoms on the leaf were examined under differential interference contrast (DIC) and epifluorescence (UV excitation filter) microscopes. Bacterial cell numbers, inspected over time, remained stable throughout the monitoring period (10^8^ CFU mL^−1^). Chlorophyll fluorescence imaging showed that *P. syringae* pv. tomato DC3000 caused visible disease symptoms on plants, with a sharp decrease observed in chlorophyll content (Figure 3). However, we found that EN21 had a protective effect on pathogen-infected plants. The EN21-treated plants appeared to be very similar to the non-infected control plants; with the aid of DIC and epifluorescence microscopy, non-infected and EN21-treated *Arabidopsis* plants, as well as those treated with the pathogen-EN21 co-culture, were observed to have more intact inner tissue and larger numbers of chloroplasts per cell than pathogen-infected plants (Figure 3). 

The chlorophyll content of *Arabidopsis* leaves is an indirect indicator of the plant’s health status, with the data presented in Table 3 demonstrating a sharp reduction in both types a and b of chlorophyll in *P. syringae* pv. tomato DC3000-infected *Arabidopsis* leaves and a partial recovery in total chlorophyll with the pathogen-EN21 co-culture. Chlorophyll levels in EN21-treated leaves were similar to those detected in control leaves.

### 3.7. EN21 Reduces P. syringae pv. Tomato Virulence in Tomato Plants

**Figure 4:**
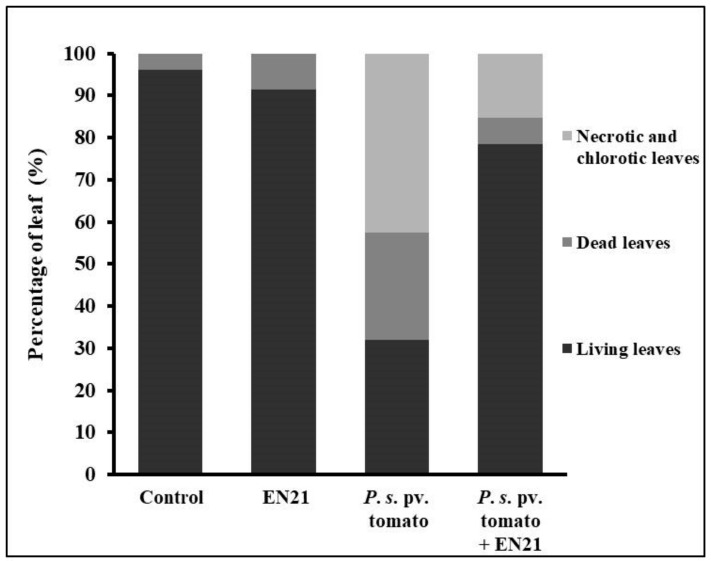
Percentage of living, dead, necrotic and chlorotic tomato leaves. Leaves were inoculated with sterile water (control), EN21 and phytopathogen mono-cultures and phytopathogen-EN21 co-culture.

Disease spread caused by *P. syringae* pv. tomato DC3000 was monitored in previously bacterised tomato plants by visually inspecting leaf tissue chlorosis at 3 dpi (Figure 4) and by measuring total plant dry weight and chlorophyll levels post treatment. The application of EN21-*P. syringae* pv. tomato DC3000 co-cultures to tomato plants revealed that strain EN21 is a promising biocontrol agent. 

The shoot, root and total dry weight of the plants is given in Table 4. Although the results indicate that the total dry weight of pathogen-infected tomato plants decreased, tomato plants treated with EN21 and the EN21-phytopathogen co-culture remained unchanged with respect to this parameter as compared to the non-infected plants. We were unable to evaluate the PGP activity of EN21 in this experiment due to the short time period (5–7 days) involved.

**Figure 5:**
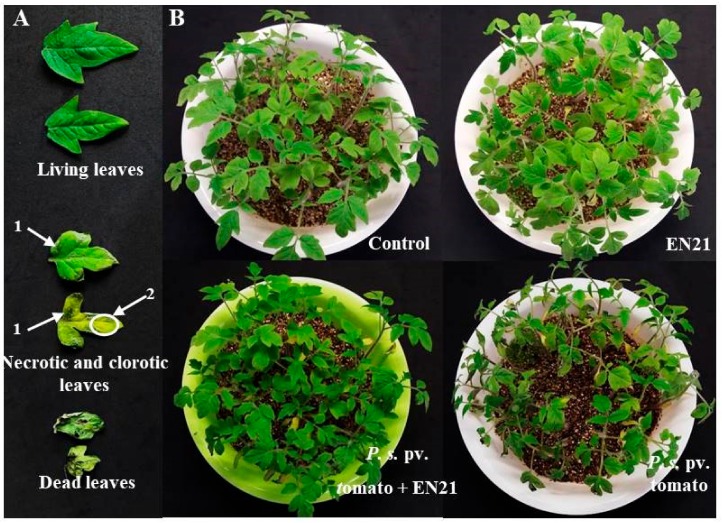
Tomato plants sprayed with sterile water (control), EN21, *P. syringae* pv. tomato and *P. syringae* pv. tomato + EN21 (**B**). Disease symptoms were inspected and photographed three days after infection (**A**). Necrotic (1A) and clorotic (2A) symptoms were observed.

Using visual monitoring, we detected symptoms of infection in leaves treated with the pathogen alone and the EN21-pathogen co-culture (Figure 5). However, severe symptoms, with large numbers of chlorotic leaves and necrotic lesions, were observed in *P. syringae* pv. tomato-infected plants. We observed a sharp reduction in dead, necrotic and chlorotic leaves treated with the EN21-pathogen co-culture, which was similar to the pattern found in EN21-treated control plants. The photographs of plants in Figure 5 show the reduction caused by EN21 in disease symptoms, which is in line with the effects previously observed in the model plant *Arabidopsis*. It also demonstrates the potential of *S. equorum* strain EN21 to be used as an effective phytopathogen biocontrol agent.

As with *Arabidopsis* plants, we observed a sharp reduction in types a and b of chlorophyll in *P. syringae* pv. tomato DC3000-infected tomato leaves, while the pathogen-EN21co-culture caused a partial recovery in total chlorophyll (Table 5). Chlorophyll levels in EN21-treated leaves were similar those observed in the controls.

## 4. Discussion

Global climate change currently affects crop growth, quality and yields which could be significantly improved by a sustainable and efficient strategy involving plant growth-promoting rhizobacteria (PGPR). 

In a previous screening of 49 soil bacteria, strain EN21, which was isolated from the halophytic plant *Salicornia hispanica*, was found to contain the most effective PGP activity. With the aid of 16S rRNA gene sequencing, EN21 was identified as belonging to the bacterial species *Staphylococcus equorum*. Although the PGP activity and biocontrol potential of bacteria such as *Pseudomonas* sp. isolated from halophytes have been extensively studied [55], very little is known about the bacterium *S. equorum*. Also, while PGP activity in the bacteria *S. cohnii*, *S. capitis* and *S. warneri* of the genus *Staphylococcus* has previously been described [56], to our knowledge, only two studies have characterized this activity in *S. equorum* strains Se1 and Se2 isolated from halophytes in *Arabidopsis thaliana*, which showed an increase in weight and root length [57,58]. 

Jeong et al. [59] have highlighted the halotolerance of *S. equorum*, which can withstand salt concentrations of 0 to 25% (*w*/*v*) NaCl, while other studies have described the safety and technological characteristics of this bacterium, which was isolated from various fermented foods. While Jeong et al. [59] found that *S. equorum* lacks functional virulence factors, our Microtox test also confirmed the non-toxic nature of strain EN21, with an EC_50_ of 125.7%.

Using biopriming studies and in vivo experiments, we evaluated the PGP activity of *S. equorum* strain EN21 in tomato plants under sterile conditions. However, plant inoculation treatment, which was insufficient on its own, needed to be combined with biopriming for the bacterium to generate significant plant growth. An increase in shoot and root dry weight and shoot length was observed in EN21-treated tomato plants as compared to controls plants. Seed biopriming with living bacterial inocula has been reported to improve harvest quality and yields [60]. Earlier studies have also shown the benefits of combining biopriming and inoculation with commercial biofertilizers containing bacterial species, such as *Pseudomonas* sp. and *Bacillus subtilis*, in plant growth promotion [61,62]. 

We investigated the strategy of combining plant growth promotion rhizobacteria with pathogen communication silencing to control bacterial plant infections, with the virulence factors of many bacterial phytopathogens having been found to be regulated by QS systems [22,23]. One promising strategy to combat infection is based on QQ signal inactivation by AHL signalling molecule degradation. We evaluated the efficiency of QS by *S. equorum* strain EN21 with respect to a wide range of synthetic AHLs and crude AHL extracts from pathogenic bacteria such as *Agrobacterium fabrum* C58 ATCC 33970^T^, *Dickeya dianthicola* DSM 18054^T^, *D. solani* IPO 2222^T^, *Erwinia amylovora* CECT 222^T^, *Pectobacterium atrosepticum* CECT 314^T^, *P. carotovorum* subsp. *carotovorum* CECT 225^T^, *Pseudomonas syringae* pv. *syringae* LMG 1247^T^ and *P. syringae* pv. tomato DC3000. AHL degradation was effective in all cases except for *D. dianthicola*. In the EN21-phytopathogen co-culture experiments, we observed an attenuation of some virulence factors. AHL interference activity has also been shown to inhibit the virulence factors of QS-regulated excreted QQ yayurea compounds A and B [15].

To our knowledge, the QQ activity of *Staphylococcus* sp. against phytopathogens has not been studied. Newman et al. [63] have reported that pathogenic bacterial virulence in plants is attenuated by this genus but by the degradation of fatty acid cell-to-cell signalling factors and diffusible signal factors (DSFs). Another study found that staphylococcal species, such as *S. delphini*, *S. intermedius* and *S. lutrae*, that excrete quorum quenching compounds called yayurea (A and B) suppress QS signalling and inhibit the growth of *P. aeruginosa* [64]. However, it found no QQ activity against *P. aeruginosa* by *S. equorum* subsp. *equorum* DSMZ 20674^T^. 

The highly effective quorum quenching of EN21 correlates with its capacity to degrade AHLs, as demonstrated by in vitro experiments on potatoes, carrots and pears to evaluate the QQ activity of EN21 in tubers and fruits. These experiments showed the effective interruption of QS by EN21 in *Dickeya solani*, *Pectobacterium carotovorum* subsp. *carotovorum* and *Erwinia amylovora*. Bacterial cell numbers were inspected over time and remained stable throughout the monitoring period, indicating that the reduction in phytopathogenic virulence by EN21 was due to effective QS interruption and not pathogen growth inhibition. Although similar results have been described with respect to other bacteria such as *Bacillus* spp., antibiotic activity can interfere with bacterial growth [65,66]. 

*In vivo* experiments with model plant *Arabidopsis thaliana* also detected EN21 quorum quenching activity against *P. syringae* pv. tomato DC3000, with chlorophyll fluorescence images of leaves showing how pathogen-infected plants are protected by EN21. DIC microscopy revealed intact inner tissue and a larger number of chloroplasts per cell in leaves treated with the pathogen-EN21 co-culture. Two studies have reported the effectiveness of measuring chlorophyll fluorescence to rapidly screen photosynthetic processes involved in the health status of plants [67,68]. Our study shows a sharp reduction in chlorophyll in *P. syringae* pv. tomato-infected *Arabidopsis* leaves and a partial recovery in total chlorophyll levels after treatment with the pathogen-EN21co-culture. 

Our in vivo experiments with *P. syringae* pv. tomato-inoculated tomato plants revealed that treatment with strain EN21 could be an ideal phytopathogen biocontrol agent. In these experiments, *P. syringae* pv. tomato-infected plants underwent an excellent recovery, as indicated by dry weight, chlorophyll levels and disease symptoms in the biocontrol assays. A sharp reduction in dead, necrotic and chlorotic leaves was observed following EN21 treatment of pathogen-infected plants. All these findings with respect to tomato plants are in line with those for the model plant *Arabidopsis thaliana*. 

Previous studies showed the reduction of soft rot symptoms produced by *Pectobacterium carotovorum* by transformed strains with an AHL-degrading gene, such as *Lysobacter enzymogenes* or *Pseudomonas putida* [69,70]. The reduction in phytopathogen virulence by QQ activity has also been studied in other bacteria such as *Pseudomonas fluorescens* [66], some of whose antibiotic production can inhibit tomato plant root development [71] and trigger antibiotic resistance in many bacterial phytopathogens. 

Little is known about the biological control potential of halotolerant bacteria based on AHL degradation. Nevertheless, the inoculation of crops with natural halotolerant PGPR strains such as *S. equorum* strain EN21, with its high quorum quenching capacity, could be an important biocontrol strategy for both saline and non-saline soils, whose principal advantages are the prevention of antibiotic resistance and use of genetically engineered bacteria. 

## Figures and Tables

**Table 1:** Effects of *S. equorum* strain EN21 on tomato seed biopriming. Length values are expressed as the mean ± SD. Increases in seedling length, germination rate and vigour index were calculated in relation to control seeds.

	Control	Biopriming with EN21
Root length (cm)	2.7 ± 1.0	2.8 ± 0.9
Root length increase (%)	-	3.9
Shoot length (cm)	2.3 ± 0.7	2.6 ± 0.8
Shoot length increase (%)	-	11.6
Total length (cm)	5.0 ± 1.6	5.4 ± 1.6
Total length increase (%)	-	7.5
Germination rate (%)	77.8	90.0
Germination rate increase (%)	-	15.7
Vigour index	390.9	486.1
Vigour index increase (%)	-	24.4

**Table 2:** Effects of inoculation treatment and biopriming with strain EN21 on tomato plants. Length and dry weight values are expressed as the mean ± SD. * indicates statistically significant differences between treated plants and control (*p* ≤ 0.05).

	Inoculation Treatment		Biopriming + Inoculation Treatment	
	Control	EN21	Control	EN21
Root length (cm)	11.9 ± 1.2	11.8 ± 1.4	12.3 ± 2.2	12.1 ± 1.7
Shoot length (cm)	18.2 ± 2.8	20.0 ± 2.1	19.3 ± 1.6	21.3 ± 1.6 *
Total length (cm)	30.1 ± 2.6	31.8 ± 1.7	31.6 ± 2.6	33.4 ± 2.7
Root dry weight (g)	0.028 ± 0.013	0.038 ± 0.012	0.017 ± 0.004	0.027 ± 0.011 *
Shoot dry weight (g)	0.121 ± 0.084	0.173 ± 0.051	0.218 ± 0.098	0.466 ± 0.071 *
Total dry weight (g)	0.149 ± 0.090	0.211 ± 0.062	0.234 ± 0.101	0.493 ± 0.080 *

**Table 3:** Chlorophyll quantification in the virulence loss test of *P. syringae* pv. tomato in *A. thaliana*.

	Chlorophyll a (μg mL^−1)^		Chlorophyll b (μg mL^−1^)		Total Chlorophyll (μg mL^−1^)	
	Arnon	Lichtenthaler	Arnon	Lichtenthaler	Arnon	Lichtenthaler
Control	1.39	1.33	0.55	0.38	1.95	1.72
EN21	1.24	1.18	0.53	0.39	1.77	1.57
*P. s.* pv. tomato	0.14	0.13	0.08	0.06	0.22	0.19
*P. s.* pv. tomato + EN21	0.34	0.32	0.23	0.18	0.57	0.51

**Table 4:** Effects of inoculation with strain EN21, *P. syringae* pv. tomato and EN21+ *P. syringae* pv. tomato co-culture on tomato plants. Dry weight values are expressed as the mean ± SD. * indicates statistically significant differences between treated plants and control (*p* < 0.05).

	Control	EN21	*P. s*. pv. Tomato	*P. s*. pv. Tomato + EN21
Root dry weight (g)	0.015 ± 0.005	0.022 ± 0.006	0.009 ± 0.003	0.016 ± 0.003
Shoot dry weight (g)	0.032 ± 0.007	0.041 ± 0.010	0.016 ± 0.005 *	0.032 ± 0.006
Total dry weight (g)	0.048 ± 0.010	0.063 ± 0.015	0.024 ± 0.008 *	0.048 ± 0.008

**Table 5:** Chlorophyll quantification in tomato plants.

	Chlorophyll a (mg g^−1^)		Chlorophyll b (mg g^−1^)		Total Chlorophyll (mg g^−1^)	
	Arnon	Lichtenthaler	Arnon	Lichtenthaler	Arnon	Lichtenthaler
Control	0.62	0.29	0.26	0.09	0.87	0.37
EN21	0.51	0.24	0.21	0.08	0.72	0.32
*P. s.* pv. tomato	0.57	0.27	0.24	0.09	0.81	0.36
*P. s.* pv. tomato + EN21	0.76	0.37	0.33	0.12	1.10	0.48

## Supplementary Materials

Supplementary materials can be found at https://www.mdpi.com/2076-2607/8/1/42/s1.
