# Supplementary material for: Silencing of Phytopathogen Communication by the Halotolerant PGPR Staphylococcus Equorum Strain EN21
Source: Microorganisms. 2019 Dec 24;8(1):42. doi: 10.3390/microorganisms8010042 (PMC7022284; doi:10.3390/microorganisms8010042)
Supplement: Supplementary file 1 [file microorganisms-08-00042-s001.pdf]

Silencing of phytopathogen communication by the halotolerant PGPR *Staphylococcus equorum* strain EN21

Clara Vega[1,2], Miguel Rodríguez[1,2], Inmaculada Llamas[1,2*], Victoria Béjar[1,2], Inmaculada Sampedro[1,2*]

[1]Department of Microbiology, Faculty of Pharmacy, University of Granada, 18071 Granada, Spain.

[2]Institute of Biotechnology, Biomedical Research Center (CIBM), University of Granada, 18100 Granada, Spain.

***Corresponding authors:**

Inmaculada Sampedro (isampedro@ugr.es) and Inmaculada Llamas (illamas@ugr.es)

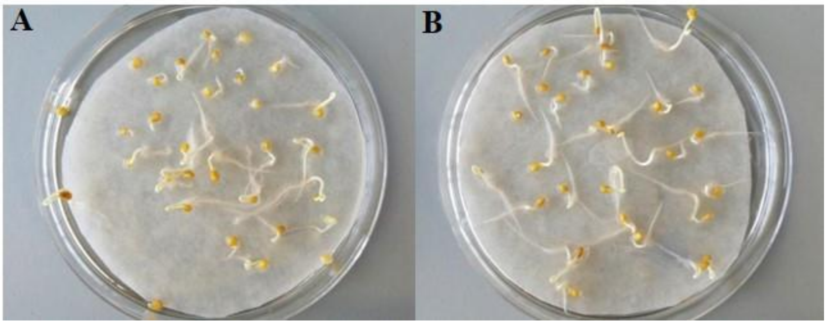

Figure S1. Effect of tomato seed biopriming with *S. equorum* strain EN21. A) Control seeds. B) EN21 bacterized seeds.

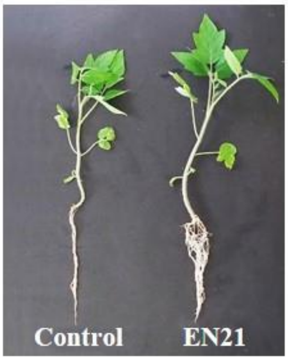

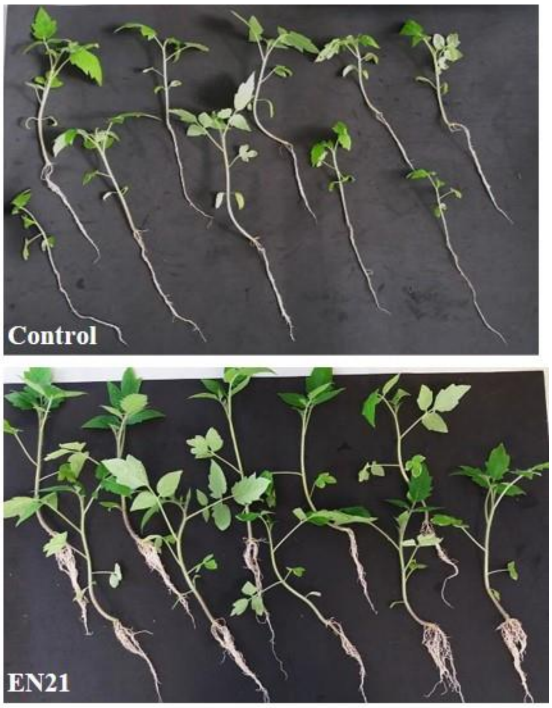

Figure S2. Effect of biopriming + inoculation treatment with EN21 strain on tomato plants.

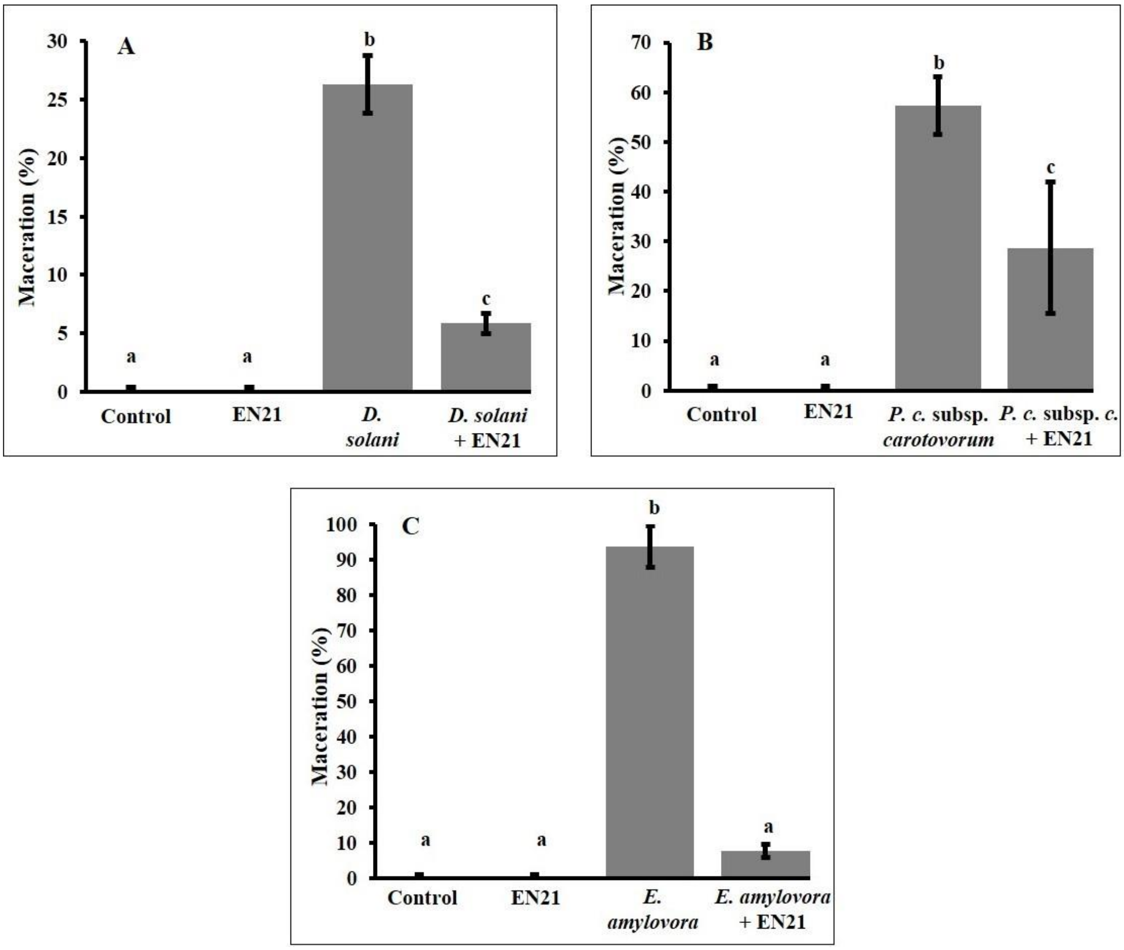

Figure S3. Maceration (%) of potato tubers (A), carrots (B) and pears (C) in *D. solani*, *P. carotovorum* subsp. *carotovorum* and *E. amylovora* mono-cultures, respectively, and in co-culture with EN21. Error bars represent the standard deviation. Letters indicate statistically significant differences ($p \leq 0.05$).

Table S1. Effect of EN21 on enzymatic activities of phytopathogens in co-cultures. (-) no activity, (+) weak activity, (++) moderate activity, (+++) strong activity. ND = not determined.

**Phenotype**	***S. equorum***	***A. fabrum***		***D. solani***		***E. amylovora***		***P. atrosepticum***		***P. c.* subsp. *carotovorum***		***P. s.* pv. *syringae***		***P. s.* pv. tomato**	
	**EN21**	Control	+EN21	Control	+EN21	Control	+EN21	Control	+EN21	Control	+EN21	Control	+EN21	Control	+EN21
**Amylase**	-	-	ND	+	+	-	ND	-	ND	-	ND	++	-	+++	+
**Caseinase**	-	+	+	++	+	-	ND	-	ND	-	ND	-	ND	+	+
**Cellulase**	+	-	ND	-	ND	-	ND	-	ND	-	ND	+	+	-	ND
**Chitinase**	-	-	ND	-	ND	-	ND	-	ND	-	ND	++	+	-	ND
**DNase**	-	++	++	++	+	-	ND	+++	++	++	++	++	++	+	+
**Phosphatase**	++	-	ND	+++	++	++	++	-	ND	++	++	++	++	+++	++
**Gelatinase**	-	+	+	+	+	-	ND	-	ND	+	+	+	-	-	ND
**Lecithinase**	-	-	ND	+++	++	-	ND	-	ND	++	+	-	ND	+	+
**Lipase Tween 20**	++	-	ND	++	++	++	++	+++	++	+++	++	++	++	++	++
**Lipase Tween 80**	-	-	ND	+	+	+	+	+	+	+	+	-	ND	+	+
**Surfactants**	+	+++	+	+	+	-	ND	-	ND	-	ND	+	+	+	+
